# Aging and gender effects in native T1 and extracellular volume fraction assessment using SASHA

**DOI:** 10.1186/1532-429X-18-S1-Q3

**Published:** 2016-01-27

**Authors:** Joseph J Pagano, Kelvin Chow, Ian Paterson, Richard B Thompson

**Affiliations:** 1 Biomedical Engineering, University of Alberta, Edmonton, AB Canada; 2 Medicine, University of Alberta, Edmonton, AB Canada

## Background

Reference values for T_1_ mapping-derived extracellular volume fraction (ECV) in healthy individuals are not currently well established. Histological measurements in autopsy studies have shown decreasing ECV with healthy aging in men, however recent non-invasive measurements of ECV using different T_1_ mapping techniques are inconsistent with respect to the effect of aging and gender, with a relatively wide range of values depending on the method. The goal of the current study was to characterize native T_1_ and ECV as a function of age in healthy individuals (no cardiovascular risk factors or medication) with the SAturation-recovery single-SHot Acquisition (SASHA) method (Magn Reson Med. 2014 Jun;71(6):2082-95), providing comparison to existing literature.

## Methods

Well characterized healthy individuals from the Alberta HEART study (BMC Cardiovasc Disord. 2014 Jul 25;14:91) underwent CMR on a Siemens 1.5T system (Sonata, Avanto) with T_1_ measurements using the SASHA pulse sequence. Imaging was performed on a mid-ventricular short-axis slice at baseline (pre-contrast) and ~15 minutes after intravenous administration of 0.15 mmol/kg gadobutrol. ECV was measured in the ventricular septum, calculated as (1-hct)*(Myocardium ΔR_1_)/(Blood ΔR_1_), where ΔR_1_ is 1/T_1_ post - 1/T_1_ pre, and hct was the most recent hematocrit.

## Results

**Figure 1:**
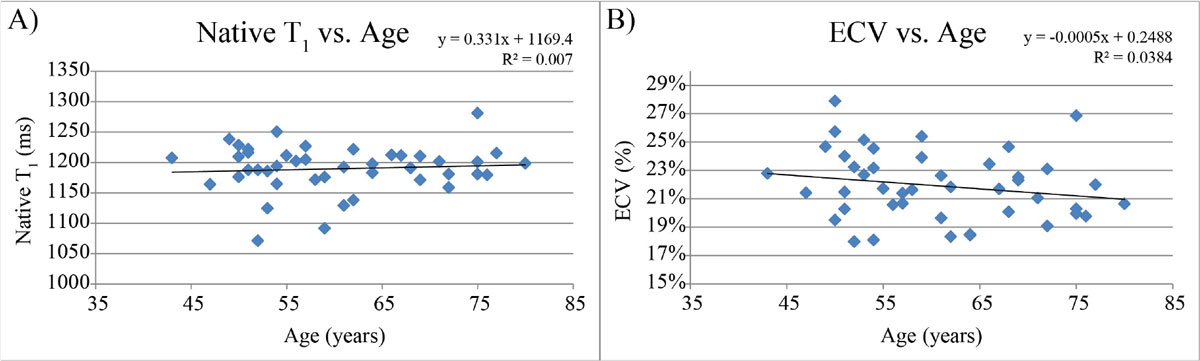
**A) SASHA native T**_**1**_ **values vs. age**. B) SASHA extracellular volume fraction (ECV) vs. age.

Native T_1_ and ECV measures were available from 44 individuals (60.7 ± 9.6 years, range 43-80, 15 male) free from cardiovascular disease, diabetes, hypertension, and not on any cardiovascular medication. Average native myocardial T_1_ value was 1189 ± 38 ms, which was increased in women compared to men (1201 ± 29 vs. 1167 ± 44 ms, p < 0.05), however did not vary significantly with age (Figure 1A; p = 0.59). Average ECV was 22 ± 2% (range 18-28%), and did not vary significantly with age (Figure 1B; p = 0.20) or gender (men: 21 ± 2% vs. women: 22 ± 2%; p = 0.14). SASHA ECV values were similar to a previous histology (p > 0.05) study. SASHA native T_1_ values were higher and SASHA ECV values were lower than inversion recovery based techniques in groups free of cardiovascular risk factors (native T_1_ comparisons only for 1.5T; p < 0.05 for all comparisons) (Table 1). Gender and age effects are noted to be different between methods (Table 1).

## Conclusions

SASHA ECV values showed no dependence on age or gender and were 14-27% smaller as compared to inversion-recovery techniques, but with good general agreement to histological studies. SASHA native T_1_ times are 19-20% longer than inversion-recovery techniques, and though they are longer in women, there is no age dependence. Significantly different ECVs by method reflect systematic differences in blood and myocardial T_1_ values (native and post-contrast), consistent with previous reports (Kellman, J Cardiovasc Magn Reson. 2014 Jan 4;16:2). Discrepancies in the relationship between native T_1_ and ECV by age and gender warrant more detailed comparison of methods as the field moves towards universal age/gender reference values.

**Table 1:** Comparison of native T1 and extracellular volume fraction between methods

Study	Technique	Field Strength	n	% Female	Age (yrs)	ECV (%)	Gender Effect	Age Effect	Native T1 (ms)	Gender Effect	Age Effect
Pagano	SASHA	1.5T	44	66	61 ± 10	22 ± 2	No effect	No effect	1189 ± 38	Female>Male	No effect
Olivetti1	Histology	N/A	67	42	63 ± 11	21 ± 4	NR	Decreases1, men only2	-	-	-
Sado3	IR single-shot FLASH EQ-CMR	1.5T	81	48	43 (24-81)	25 ± 4	Female>Male	No effect	NR	NR	NR
Neilan4	Cine Look-Locker	3T	32	56	49 ± 15	28 ± 3	No effect	Increases	NR	NR	NR
Liu5	MOLLI	1.5T	235	39	65 ± 8	NR	No effect	No effect	NR	No effect	No effect
Dabir6	MOLLI	1.5T	34	NR	NR	25 ± 4	No effect	No effect	950 ± 21	No effect	No effect
Dabir6	MOLLI	3T	32	NR	NR	26 ± 4	No effect	No effect	1052 ± 23	No effect	No effect
Fontana7	ShMOLLI	1.5T	50	47	47 ± 17	27 ± 3	NR	NR	NR	NR	NR
Piechnik8	ShMOLLI	1.5T	342	51	38 ± 15	NR	NR	NR	962 ± 25	Female>Male	Decreases in women

1-Results adapted from Figure 3; Olivetti, Circ Res. 1991 Jun;68(6):1560-8

2-Olivetti, J Am Coll Cardiol. 1995 Oct;26(4):1068-79

3-Sado, Heart. 2012 Oct;98(19):1436-41

4-Neilan, JACC Cardiovasc Imaging. 2013 Jun;6(6):672-83

5-Liu, J Am Coll Cardiol. 2013 Oct 1;62(14):1280-7

6-Dabir, J Cardiovasc Magn Reson. 2014 Oct 21;16:69

7-Fontana, J Cardiovasc Magn Reson. 2012 Dec 28;14:88

8-Piechnik, J Cardiovasc Magn Reson. 2013 Jan 20;15:13

NR = Not Reported

